# Investigation of high school students’ social emotional learning skills and social media use

**DOI:** 10.3389/fpsyg.2024.1425497

**Published:** 2025-01-06

**Authors:** Harun Şahin, Meriç Eraslan, Muhammed Ali Özkan

**Affiliations:** ^1^Department of Curriculum and Instruction, Akdeniz University Institute of Educational Sciences, Antalya, Türkiye; ^2^Department of Physical Education and Sports Education, Akdeniz University Faculty of Sport Science, Antalya, Türkiye; ^3^Doctoral Program in Curriculum and Instruction, Akdeniz University Institute of Educational Sciences, Antalya, Türkiye

**Keywords:** social emotional learning, social media, adolescence, high school, student

## Abstract

The current study aims to examine the association between high school students’ social–emotional learning (SEL) skills and their use of social media, as well as to explore potential variations based on certain variables. The research utilized relational and comparative survey methodologies, with 325 high school students participating. Data were gathered through the administration of the “Social Emotional Learning Scale” and the “Social Media Use Scale.” Analytical techniques such as *t*-tests, ANOVA, and Pearson correlation coefficient were applied to analyze the collected data. The results indicate a slight, adverse connection between the social–emotional learning abilities of students and their utilization of social networking sites. Furthermore, the research revealed that the average ratings for both social–emotional learning abilities and social media usage were moderate. Additionally, no significant differences were observed in social–emotional learning skills and social media use based on gender or grade level.

## 1 Introduction

The 21st century is an age in which humanity has made great leaps in every field, and technology, and therefore information and communication tools, has rapidly changed people and social structure (İşeri, 2016). Many developments in this century have also affected the education system (Körler, 2011). Today, what is expected from a school is to educate students as responsible, healthy and academically, socially and individually qualified individuals. In this context, schools should include systematic programs that will ensure the development of students in all aspects (Greenberg et al., 2003). Since the 1980s, it has been accepted that academic intelligence alone will not be sufficient in education, and concepts such as multiple intelligence, emotional intelligence, character education, moral education have started to gain importance in education (McKenzie, 2004).

The social needs of individuals differ in each developmental period. For example, the most important social qualities that should be acquired by the individual at school age are gaining personal independence, spending time in harmony with their peers and learning gender roles (Gardiner and Gander, 1993). Individuals’ social needs vary throughout their development. For instance, the significance of friendship relationships is substantial in the social development of high school students. The process whereby individuals in their high school years enhance their social skills in interactions with peers is an integral part of their personal development (Brown, 2004). This process plays a critical role in adolescents’ identity formation and understanding of social roles. To create the desired student profile in education, developing social–emotional learning skills is crucial for all students, irrespective of their specific needs, especially in the adolescence period due to the developmental characteristics of this period (Ağırkan, 2021). Adolescence is a period in which hormonal changes, emotional transitions and social interactions are experienced together with rapid physical growth in the individual (Santrock, 2016). It is stated that the individual seeks identity, attaches more importance to social relations, brings emotionality to the fore, is open to new experiences, makes important plans for the future and attaches greater importance to the sense of independence (Gardiner and Gander, 2007). In this period of their life, individuals try to act independently from their parents and try to socialize with their friends and peers. Adolescents have the opportunity to acquire numerous social–emotional skills through the social relationships they form with their peers (Çiftçi, 2018a). Risk conditions such as violence, alcohol consumption, smoking, depressive symptoms, communication problems, lack of problem solving competence, academic failure, absenteeism rates, school dropouts that occur during adolescence are important problem areas that should be addressed (Ağırkan, 2021). To avoid these issues, it is crucial for individuals to cultivate traits such as tolerance, empathy, the ability to form healthy relationships, sound decision-making skills, the development of moral values, emotional management, career planning, and the cultivation of self-esteem and social awareness (Bahçuvanoğlu, 2019). An essential factor in individuals’ skill development is social and emotional learning (Cohen, 1999; Elias et al., 2003; Elliot and Busse, 1991).

Albert Bandura, a pioneer of Social Learning theory, played a key role in shaping social–emotional learning programs and establishing their foundation (Candan, 2018). Social emotional learning studies were first systematically initiated by the Fetzer Institute in 1994. According to this, while increasing academic success is emphasized in the social emotional learning program, attention is also drawn to the underlying causes of the problem behaviour, rather than just focusing on a certain behavior. Another organization, the Collaborative for Academic, Social, and Emotional Learning (CASEL), underscores the importance of social–emotional learning from preschool to high school (Greenberg et al., 2003). Founded by Goleman in 1994, CASEL is a scientific organization that strives to develop social emotional learning to the highest level, both theoretically and practically. The goal is to foster the healthy development and well-being of children and teenagers through education, by incorporating research-based social–emotional learning programs from preschool through high school (CASEL, 2005).

Social–emotional learning involves recognizing and regulating emotions, establishing constructive objectives, empathizing with others, maintaining relationships, and making prudent choices (Pikul, 2015). Elias (2006) defines social emotional learning as being able to understand and to be aware of one’s own and others’ feelings, to communicate with other individuals and to express their own feelings and thoughts, to know their needs and to realize their strengths and weaknesses. Social–emotional skills, which refer to an individual’s awareness of his/her own feelings and thoughts, managing his/her own emotions, empathizing, building relationships and making effective decisions, have an important place in our lives in order to be a good student, a good employee and a good citizen (CASEL, 2013). Social–emotional learning instructs individuals to identify, structure, and articulate the social and emotional facets of their lives, empowering them to effectively manage their life responsibilities (Norris, 2003).

Education aims to equip students with essential life skills across physical, cognitive, social, and emotional domains, enabling effective interaction with their social environment. This goes beyond academic achievements, emphasizing the structuring of social relations and emotional development (Ağırkan, 2021). Several programs have been developed to reduce students’ engagement in drug use, violence, and high-risk sexual behaviors in schools (Payton et al., 2000). Social and emotional learning programs are designed to assist individuals in overcoming these challenges by enhancing adolescents’ skills and mindsets, fostering resilience, and promoting a positive school climate (Yeager, 2017).

Effective schools go beyond academic preparation; they also equip students for life beyond school by integrating social and emotional learning skills with academic success (Zins and Elias, 2006). An example can illustrate the development of students’ social–emotional learning skills. The student starts the math lesson and feels disappointed because he/she does not understand a mathematical concept. This self-awareness positively affects the process of responsible decision-making. He/she takes deep breaths to relax as he/she is waiting for his/her turn to ask. This supports his/her self-regulation skill. He/she gets help from his/her teacher or classmates. This contributes to his/her social awareness and relationship management skills. He/she plans to complete the project at home in the evening. This helps him/her in the responsible decision making process (Haney, 2013). Totan (2011) argues that schools should be institutions that are open to a transformation from being an institution that only provides academic skills and knowledge to an institution supporting social and emotional learning. In preparing individuals for the future, it’s crucial to equip them with academic, emotional, and social skills (Poplawski, 2021).

The literature highlights numerous benefits of social–emotional learning for students. Adolescent relationships, particularly with friends, are recognized for fostering the development of these skills (Çiftçi, 2018b). In another study conducted on participants aged 12–15, it was understood that peer relationships were more positive as individuals’ social emotional learning skills improved (Bozkurt Yükçü et al., 2021). Research shows that social–emotional learning boosts academic performance, improves social behavior and relationships, reduces behavioral issues and psychological distress, and enhances success in university, work, family, and society (Elias, 2014; Jones and Kahn, 2017). Gargan (2017) discovered that a social–emotional learning program positively influenced school climate, improving safety, teaching quality, institutional environment, and relationships. Kothari and Wesley (2020) found that a social–emotional learning intervention significantly impacted emotional intelligence scores in adolescents. Koruklu et al. (2017) concluded that the rate of aggressive behavior is low in individuals with high social–emotional learning skills and that such individuals prefer constructive problem-solving methods in conflict resolution. Çelik (2015) discovered that high school students who develop social–emotional learning skills experience reduced educational stress. In their study, Zins et al. (2007) presented a clear, evidence-supported case indicating that social emotional learning promotes academic learning.

On the other hand, the socialization patterns of adolescents have shifted with advancements in information technologies, particularly with the significant role of online social media platforms (Bilgin et al., 2020). Social networking sites are electronic environments where large audiences can interact with each other and which are set up for people to create a social environment (Dal and Dal, 2014). In the information society, where communication, cooperation and sharing gain importance, social media plays an important role in meeting these needs. Social media has become integral to personal and social life, influencing individuals’ lifestyle choices through its various opportunities (Şişman Eren, 2014). In the adolescence period, when individuals’ needs for making friends, socializing and individualization are quite high, social media platforms virtually satisfy these needs of individuals (Atın, 2022).

Social media is a rapidly expanding global phenomenon, with the number of users projected to reach 4.4 billion by 2025, representing approximately half of the world’s population (Statista, 2022). Social media plays a central and active role in today’s social life, hosting numerous events, news, and announcements from start to finish (Çakmak, 2018). Now, individuals can easily follow political, social, cultural, economic, artistic, etc. information and agenda from the Internet and social media. Research data shows that young people reach the information they need or the information they acquire on any subject from the Internet by 90 percent and Facebook by 70 percent (Kamiloğlu and Yurttaş, 2014).

Internet use is increasing with each day depending on technological developments and it turns into addiction, especially among adolescents, and becomes a risk factor (Akın et al., 2015). As social media platforms have become an integral part of daily life, it is increasingly difficult to determine if people are addicted to social media (Andreassen and Pallesen, 2014). This makes it challenging to answer questions like, “How many hours of social media use per day lead to addiction?” Social media users do not encounter addiction issues as long as they maintain control. However, when social media usage negatively impacts daily life or health and continues despite these effects, addiction arises (Andreassen, 2015; Griffiths, 2000). Adolescents’ desire to be popular with their social media posts is effective in their more intense use of the internet (Kaygusuz, 2013). The negative messages that social media constantly gives to people encourage children for violence, substance use and sexual life for which they are not yet mentally and physically ready (Kids Health, 2004). Factors contributing to social media addiction suggest that adolescents should engage in activities that allow them to express themselves more freely. Instead of prohibition, it is essential to guide them in the safe use of social media (Güney and Taştepe, 2020).

Delving into the literature, we find both positive and negative impacts of social media on individuals. For adolescents, longer social media use negatively affects academic success and friendships (Kumcağız et al., 2019). Çakmak (2014) concluded that people with a low frequency of social media use have high communication anxiety in a study conducted with the participation of 1,139 people using face-to-face interview method. Demir’s (2016) study found that daily social media users express themselves better in that environment compared to those who use it no more than twice a week. Yanar (2015) on the other hand concluded that the rate of alienation from face-to-face relationships is high for those who spend time on social media. Sistek Chandler (2012) outlined the positive aspects of social media, including its ability to help shy students engage with others, build positive peer relationships, and facilitate idea generation and sharing.

No previous study has compared the extent of social media usage and social–emotional learning skills. Existing studies on their relationship tend to focus on social media addiction rather than mere use. It is deemed crucial to comprehend the link between social–emotional learning skills, vital for students’ social and emotional growth, and the growing prevalence of social media. Understanding the interaction between students’ social and emotional development and social media is considered an important research area in today’s technology-driven communication environment. These concepts, deemed critical in today’s evolving social landscape due to the internet and technological advancements, are believed to be interconnected.

This study investigates how high school students’ social–emotional learning skills relate to their social media use and whether this connection varies based on specific variables.

1. What is the level of the relationship between high school students’ social emotional learning skills and social media use?
2. What is the level of high school students’ social emotional learning skills?
3. What is the level of high school students’ social media use?
4. Do high school students’ social emotional learning skills vary significantly depending on gender?
5. Does high school students’ social media use vary significantly depending on gender?
6. Do high school students’ social emotional learning skills vary significantly depending on grade level?
7. Does high school students’ social media use vary significantly depending on grade level?

## 2 Method

### 2.1 Research model

This research employs a survey model and falls under the category of descriptive studies. Descriptive studies seek to elucidate the interplay of circumstances, considering the connection between present occurrences and past situations and conditions (Kaptan, 1989). Descriptive studies examine events in their natural environments (Kaptan, 1973). This study used a survey model to examine the correlation between high school students’ social–emotional learning skills and their social media use, as well as to determine if this relationship varies significantly based on certain variables. The study used relational and comparative survey models. The relational model aims to explore connections between two or more variables (Karasar, 2017). Relational studies aim to identify relationships between variables and understand potential cause-and-effect relationships, while comparative survey models focus on differences among groups without intervening in circumstances or participants (Büyüköztürk et al., 2015). The comparative model in this study examines differences based on gender and grade level (Karasar, 2017).

### 2.2 Population and sample

The study included 352 high school students (164 females, 188 males) from a public high school in Muş Province, selected using convenience sampling. In the convenience sampling method, the participants are reached starting from the participants who are easiest to reach until the target number of participants has been reached (Büyüköztürk et al., 2015). The study included 352 high school students, with 95 in 9th grade, 70 in 10th grade, 88 in 11th grade, and 99 in 12th grade. Of these, 164 were female and 188 were male.

### 2.3 Data collection tools

#### 2.3.1 Social Emotional Learning Scale

The research evaluated high school students’ social–emotional learning levels utilizing Totan’s (2018) scale, comprising 23 items and five sub-dimensions: self-awareness, social awareness, self-regulation, relationship building, and responsible decision-making. The scale, a five-point Likert scale, was designed to gauge adolescents’ social, emotional, academic development, and self-awareness levels. It showed good reliability with a Cronbach’s *α* coefficient of 0.92 for the whole scale and 0.70 to 0.83 for the sub-dimensions. Criterion-related validity was established with a correlation of 0.47 with Kabakçı and Korkut Owen’s (2010) scale and 0.44 with Coryn et al.'s (2009) scale. Test–retest reliability was also good at 0.82.

In this study, the Social Emotional Learning Scale’s validity and reliability were reevaluated, with the results detailed below.

**Figure 1:**
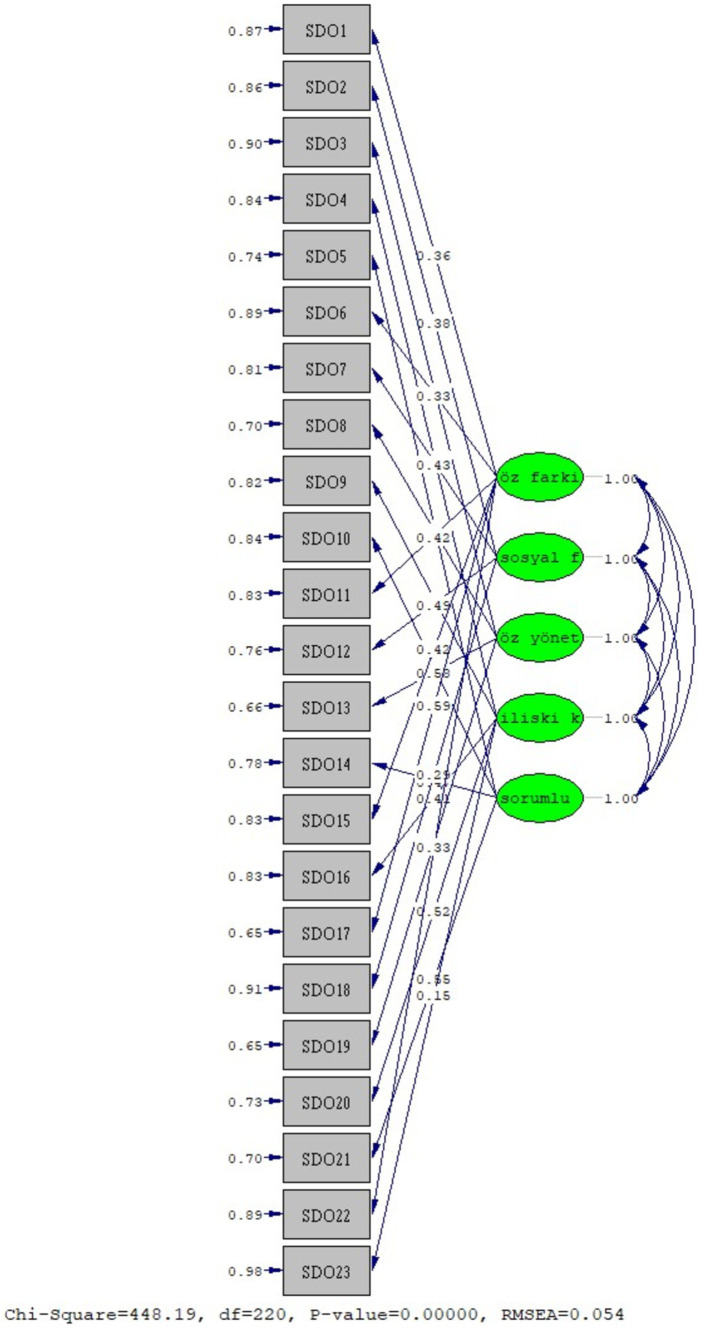
CFA model of the Social Emotional Learning Scale.

Confirmatory Factor Analysis: Figure 1 displays the results of the confirmatory factor analysis, verifying the consistency of the factor structures of the Social Emotional Learning Scale for this study.

Confirmatory factor analysis was conducted on the Social Emotional Learning Scale, yielding the following fit indices: χ^2^ = 448.19/(df = 220) = 2.04 (*p* = 0.00), RMSEA = 0.05, CFI = 0.94, GFI = 0.90, NNFI (TLI) = 0.93, and SRMR = 0.06. These values indicate a good fit for the scale (Baumgartner and Homburg, 1996; Bentler, 1980; Browne and Cudeck, 1993; Kline, 2011). Additionally, the *t*-values derived from the model confirm the significance of the factor loadings.

Reliability of the Social Emotional Learning Scale was assessed using Cronbach’s Alpha coefficient, yielding values of 0.83 for the overall scale, and 0.76, 0.79, 0.78, 0.79, and 0.75 for its sub-dimensions “Self-Awareness,” “Social Awareness,” “Self-Regulation,” “Relationship Building,” and “Responsible Decision-Making,” respectively. These results indicate that the scale and its sub-dimensions are reliable (Büyüköztürk, 2017).

#### 2.3.2 Social Media Use Scale

In this study, the “Social Media Use Scale (SMUS)” developed by Deniz and Tutgun Ünal (2019) was employed, with permission from the authors, to assess students’ social media usage. The scale comprises two sub-dimensions: “Continuity” and “Competence.” The continuity sub-dimension evaluates the extent to which an individual maintains consistent use of social media. The competence sub-dimension measures how competent a person sees himself or herself in social media. Each subscale contains 4 items, contributing to a total of 8 items in the scale, which assesses an individual’s level of social media use. The items 1, 2, 3, 4 in the scale measure continuity, while the items 5, 6, 7, and 8 measure competence. The scale uses a five-point Likert scale ranging from “Not suitable for me at all” to “Very suitable for me.” The scale has no items with reverse scoring and has a Cronbach’s *α* coefficient of 0.82 for internal consistency.

For the current study, the validity and reliability analyses of the Social Media Use Scale were made again and the results are given below.

##### 2.3.2.1 Confirmatory factor analysis

**Figure 2:**
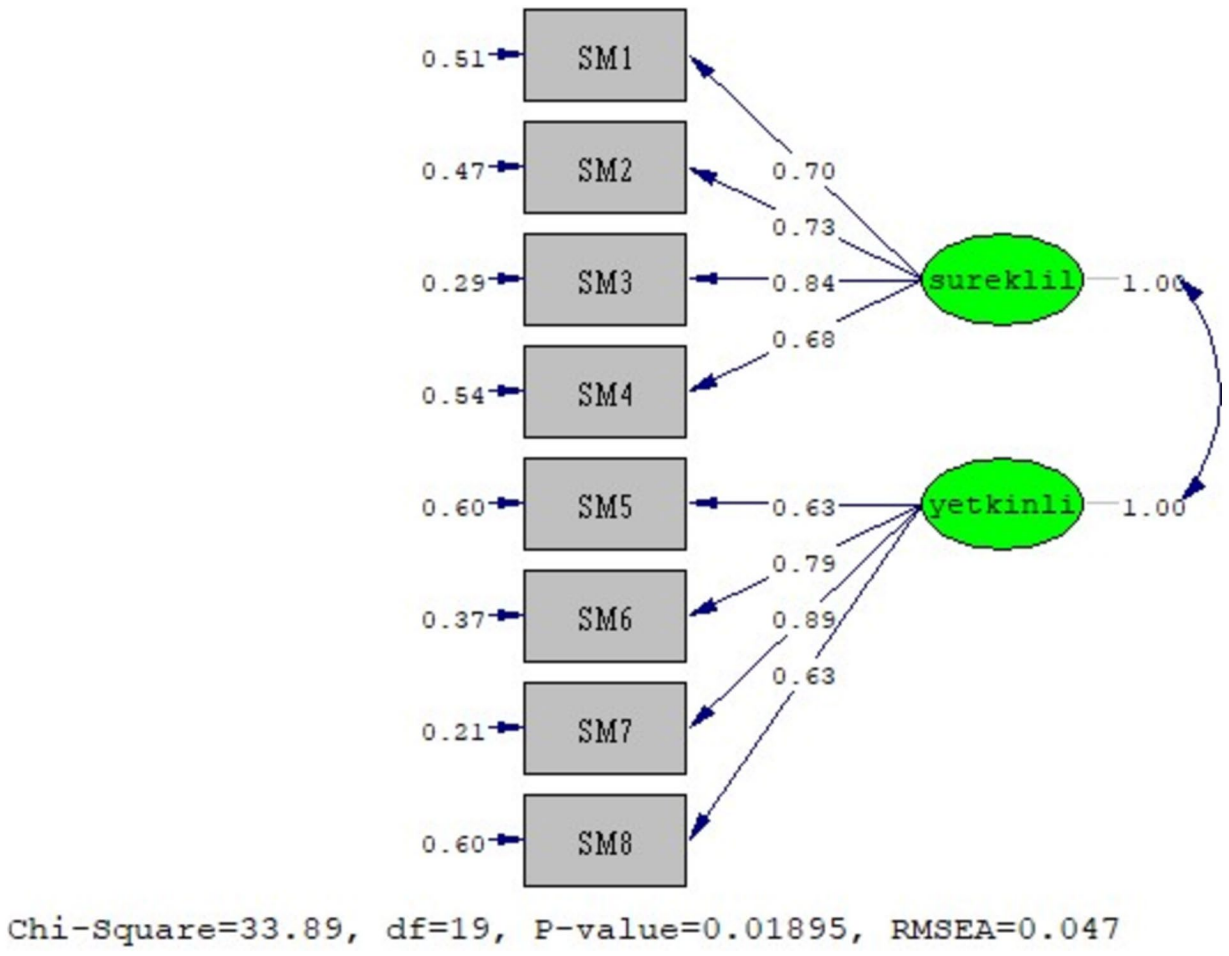
CFA model of the Social Media Use Scale.

Figure 2 presents the outcomes of the confirmatory factor analysis, which was conducted to verify if the factor structures of the Social Media Use Scale remained consistent for this study.

Confirmatory factor analysis was performed for the Social Media Use Scale, and the following values were obtained for the fit indices: (χ^2^ = 448.19/sd = 220) = 1.78 (*p* = 0.00), RMSEA = 0.05, CFI = 0.99, GFI = 0.98, NFI = 0.99, NNFI (TLI) = 0.99 and SRMR = 0.03. These values mean that the scale shows a perfect fit (Baumgartner and Homburg, 1996; Bentler, 1980; Browne and Cudeck, 1993; Kline, 2011). In addition, the *t*-values obtained from the model also confirm the significance of the factor loadings.

##### 2.3.2.2 Reliability analysis

The reliability of the Social Media Use Scale was evaluated with Cronbach’s Alpha coefficient, yielding 0.89 for the overall scale, 0.79 for the “continuity” sub-dimension, and 0.75 for the “competence” sub-dimension. These findings suggest that the scale and its sub-dimensions are reliable (Büyüköztürk, 2017).

### 2.4 Data analysis

To assess the appropriateness of the Social Emotional Learning Scale and Social Media Use Scale for the study group, confirmatory factor analysis was performed using the LISREL 8.7 program. Statistical analysis of the gathered data was carried out using SPSS 24. Normality tests were conducted for continuous data, indicating a normal distribution and justifying the use of parametric tests.

The study presents descriptive and comparative statistics for the responses obtained from the measurement tools. An independent samples *t*-test was conducted to examine gender differences in social–emotional learning skills and social media use. ANOVA was used to explore potential grade-level disparities in these skills and usage. Pearson’s Correlation Coefficient was computed to assess the relationship between social–emotional learning skills and social media use.

### 2.5 Limitations

1. The research was conducted on high school students from a specific region and under specific conditions. Therefore, generalizing the findings to the overall high school student population may be limited.
2. The data were obtained through surveys based on student self-reports. Subjective approaches or response tendencies of the students could have influenced the results.
3. The study did not control for potential influencing variables such as socio-economic status or family dynamics. The impact of these variables on the results should be considered.
4. The reliability and validity limits of the scales used (Social–Emotional Learning Scale and Social Media Use Scale) could have affected the interpretation of the results.

## 3 Results

In this section, we present the findings regarding students’ social emotional learning skills and their use of social media.

### 3.1 What is the level of the relationship between high school students’ social emotional learning skills and social media use?

Table 1 illustrates a significant yet weak negative correlation between high school students’ social–emotional learning skills and their social media use (*r* = −0.165, *p* < 0.01), suggesting that as students’ social–emotional learning skills enhance, their social media usage declines.

**Table 1:** The relationship between social emotional learning skills and social media use.

		Social emotional learning	Social media use
Social emotional learning	*r*	1	−0.165 (**)
	*p*		0.002
	*n*	352	352

^**^Significant at the level of 0.01 (*p* < 0.01).

### 3.2 What is the level of high school students’ social emotional learning skills?

As shown in Table 2, high school students’ mean scores on the sub-dimensions of the Social Emotional Learning Scale range from 3.51 to 3.87. The general mean score was found to be 3.68. According to these mean scores, although there is no clear difference, it is seen that the students are most successful in the sub-dimension of responsible decision making (3.87) and the least successful in the sub-dimension of relationship-building (3.51). The overall mean score (3.68) from the scale indicates a moderate level of social–emotional learning among students.

**Table 2:** Students’ state of using social emotional learning Skills.

Social emotional learning	*n*	x̄	ss
Self-awareness	352	3.75	0.644
Social awareness	352	3.56	0.779
Self-regulation	352	3.71	0.776
Relationship building	352	3.51	0.709
Responsible decision-making	352	3.87	0.718
Total	352	3.68	0.550

### 3.3 What is the level of high school students’ social media use?

Table 3 displays the mean scores of high school students across the sub-dimensions of the Social Media Use Scale, ranging from 2.50 to 2.57, with an overall mean score of 2.53. The highest mean score, 2.57, was observed in the competence sub-dimension, indicating a moderate level of social media use among students.

**Table 3:** Students’ state of social media use.

Social media use sub-dimensions	*n*	x̄	ss
Continuity	352	2.50	1.156
Competence	352	2.57	1.164
Total	352	2.53	1.086

### 3.4 Do high school students’ social emotional learning skills vary significantly depending on gender?

Table 4 shows that, except for self-awareness, there are no notable variances in mean scores among the sub-dimensions of the social–emotional learning skills scale and the overall scale based on gender. Female students scored significantly higher in self-awareness (x̄ = 3.84; ss = 0.624) compared to male students (x̄ = 3.68; ss = 0.655) (*p* < 0.05; *p* = 0.020). Overall, female students scored slightly higher than male students, although not significantly different, indicating similar social–emotional learning skills levels.

**Table 4:** *t*-Test findings of social emotional learning in terms of gender.

Social emotional learning	Gender	*n*	x̄	ss	sd	*t*	*p*
Self-awareness	Female	164	3.84	0.624	349	2.342	0.020
	Male	187	3.68	0.655			
Social awareness	Female	164	3.62	0.773	349	1.212	0.226
	Male	187	3.51	0.783			
Self-regulation	Female	164	3.67	0.758	349	−0.901	0.368
	Male	187	3.74	0.793			
Relationship building	Female	164	3.52	0.741	349	0.303	0.762
	Male	187	3.49	0.684			
Responsible decision-making	Female	164	3.93	0.735	349	1.524	0.128
	Male	187	3.81	0.703			
Total	Female	164	3.71	0.550	349	1.109	0.268
	Male	187	3.65	0.551			

Significance level: *p* < 0.05.

### 3.5 Does high school students’ social media use vary significantly depending on gender?

Table 5 shows a non-significant difference in social media use scores between male and female students (*p* > 0.05; *p* = 0.392), with males scoring slightly higher on average. Therefore, it can be concluded that male students’ social media use (x̄ = 2.48; sd = 1.128) is similar to that of female students (x̄ = 2.58; sd = 1.041). Moreover, male students’ mean scores in the continuity and competence sub-dimensions are slightly higher than those of female students, yet this difference lacks statistical significance. Consequently, male and female students exhibit similar patterns in their social media use.

**Table 5:** *t*-Test findings of social media usage in terms of gender.

Social media use sub-dimensions	Gender	*n*	x̄	ss	sd	*t*	*p*
Continuity	Female	164	2.49	1.139	349	−0.180	0.857
	Male	187	2.51	1.175			
Competence	Female	164	2.48	1.096	349	−1.425	0.155
	Male	187	2.65	1.217			
Total	Female	164	2.48	1.041	349	−0.857	0.392
	Male	187	2.58	1.128			

Significance level: *p* < 0.05.

### 3.6 Do high school students’ social emotional learning skills vary significantly depending on grade level?

ANOVA was utilized to determine significant differences in high school students’ social–emotional learning skills across various grade levels. The mean scores for self-awareness (*F* = 1.065; *p* > 0.05), social awareness (*F* = 0.368; *p* > 0.05), self-regulation (*F* = 0.757; *p* > 0.05), and responsible decision-making (*F* = 0.245; *p* > 0.05) did not show significant variations based on grade level. Additionally, the overall scale scores did not significantly differ across grade levels (*F* = 0.747; *p* > 0.05) (Table 6).

**Table 6:** ANOVA findings of social emotional learning skills in terms of grade level.

Social emotional learning sub-dimensions	Source of the variance	Sum of squares	sd	Mean square	*F*	*p*	Significant difference
Self-awareness	Between-groups	1.326	3	0.442	1.065	0.364	-
	Within-group	144.447	348	0.415			
Social awareness	Between-groups	0.675	3	0.225	0.368	0.776	-
	Within-group	212.830	348	0.612			
Self-regulation	Between-groups	1.373	3	0.458	0.757	0.519	-
	Within-group	210.462	348	0.605			
Relationship building	Between-groups	5.804	3	1.935	3.938	0.009	11th grade-10th grade, 11th grade-12th grade
	Within-group	170.972	348	0.491			
Responsible decision-making	Between-groups	0.383	3	0.128	0.245	0.865	-
	Within-group	180.967	348	0.520			
Total	Between-groups	0.681	3	0.227	0.747	0.525	-
	Within-group	105.761	348	0.304			

Significance level: 0.05 < *p*.

The mean score from the relationship building sub-dimension exhibited a significant difference based on grade level (*F* = 3.938; *p* < 0.05). To further investigate this difference, Tukey’s test was conducted as a post-hoc analysis. Results indicated a significant difference favoring 11th-grade students over 10th-grade students, as well as over 12th-grade students in terms of relationship building scores.

### 3.7 Does high school students’ social media use vary significantly depending on grade level?

ANOVA was used to analyze potential differences in high school students’ social media usage across various grade levels. The results indicated that the mean scores for continuity (*F* = 0.243; *p* > 0.05) and competence (*F* = 0.434; *p* > 0.05) sub-dimensions did not significantly vary based on grade level. Similarly, there was no significant difference in the mean scores for the overall scale across different grade levels (Table 7).

**Table 7:** ANOVA findings of social media usage in terms of grade level.

Social media use sub-dimensions	Source of the variance	Sum of squares	sd	Mean square	*F*	*p*	Significant difference
Continuity	Between-groups	0.982	3	0.327	0.243	0.866	-
	Within-group	468.860	348	1.347			
Competence	Between-groups	1.775	3	0.592	0.434	0.729	-
	Within-group	474.295	348	1.363			
Total	Between-groups	1.127	3	0.376	0.316	0.814	-
	Within-group	413.094	348	1.187			

Significant difference: 0.05 < *p*.

## 4 Discussion

The study investigated the correlation between high school students’ social emotional learning skills and their social media usage. Additionally, it explored these skills and media habits in relation to various variables. According to the results;

High school students’ social emotional learning skills are negatively correlated with their use of social media, albeit weakly. According to this result, as students’ social media use increases, their social emotional learning skills decrease. Such a direct relationship has not been found in the literature. In the literature, it is seen that social media addiction has been mostly investigated rather than social media use. Tutgun Ünal (2015) found that as social media usage duration increases, so does social media addiction, suggesting these terms can be used interchangeably. Atın’s (2022) study concluded that adolescents’ social media addiction levels are inversely proportional to their social–emotional learning skills. This result supports the current study. Although not directly related to the current study, there are studies on different concepts. Er Dalma (2019) found that as 5th and 6th grade students used smartphones more, their social emotional adaptation and social life skills decreased. Tilki (2021) discovered a notable difference in middle school students’ social emotional learning skills based on their digital game playing time. Accordingly, as the playing time decreases, the mean values of social emotional learning skills increase. Overall, this finding suggests that continuous social media usage may adversely affect students’ development of face-to-face communication skills, empathy, and social awareness. The intensity of digital interactions could hinder students’ abilities for deep thinking and emotional skill development.

The study concluded that high school students possess a moderate level of social emotional learning skills. Bayram (2022) conducted research with social studies teachers and discovered that their scores on the Social Emotional Learning Scale-Young Adulthood Form, both total and in sub-dimensions, ranged from 3.40 to 4.19. Therefore, social studies teachers demonstrate high social emotional learning skills based on their scores on the overall scale and its sub-dimensions. Esen Aygün and Şahin Taşkın (2017) found that students were at a moderate level in self-confidence and impulse control sub-dimensions, but at a high level in success, continuity, friendship relations, friendship perception, and self-regulation sub-dimensions, as well as in the overall scale. In addition to all these studies, certain research suggests that various programs could be effective in further enhancing these skills among students (Esen Aygün and Şahin Taşkın, 2022). Therefore, more research is needed to investigate the importance and impacts of social–emotional learning programs at the high school level within this context.

The study found that high school students’ use of social media is moderate, indicating they use it at a moderate level. With the rapid changes in technological devices, the rate of people reaching and using social media has increased, but it is seen that it has become widespread especially among adolescents (Bilgin et al., 2020). The literature reveals numerous studies on students’ use of social media and the internet. Akyürek (2020) concluded that the rate of students who use social media “more than once a day” is the highest with 60.4%. Güney and Taştepe (2020) concluded that adolescents tend to have a moderate level of social media addiction based on their research. Altunbaş and Kul (2015) discovered that 75.2% of university students continuously use social media in their study on the frequency of social media usage among university students. Koçak (2012) found that the most commonly used social media platform is social networking sites, with the age group of 15–24 being the most frequent users. Tutgun Ünal and Deniz (2020) found that social media use of generations is at a medium level. Dinçer and Kilinç (2019) examined the social media usage of 262 students at the School of Physical Education and Sports, finding that their usage level was moderate. In their study, Bodur and Korkmaz (2017) found that theology students’ use of social media is slightly above the average. Kamiloğlu and Yurttaş (2014) found in their study that almost all high school students have Facebook accounts, young people visit Facebook twice a day or more, and stay more than 1 h in each visit. Studies show that students’ social media use is generally high. This study suggests that the students’ moderate use of social media may be attributed to limited internet and technology access, given that the school offers bus-based education and the students are from rural areas.

Except for self-awareness, there was no gender difference in social–emotional learning skills among students. This suggests that female students have higher self-awareness than male students. Overall, the social–emotional learning skills of female and male students are similar. This result is supported by existing literature. Aksoy (2020) conducted a study on high school students and found that their social emotional learning levels did not significantly differ based on gender. Bahçuvanoğlu (2019) found no significant difference in the social–emotional learning skills between female and male students in the pre-test results. Similarly, Kothari and Wesley (2020) found no significant difference in the social–emotional learning levels based on gender in their study on adolescents. In the study conducted by Soylu (2007), according to the results related to the findings on the gender variable, there was no great difference between male and female students in the degree of having social and emotional skills. Yet, upon examining the means, it was evident that female participants exhibited stronger social and emotional learning skills. Unlike the present study, other studies have found a significant gender-based difference in students’ social and emotional learning skills. Kuyulu (2015) reported that female students exhibited significantly higher levels of social–emotional skills than male students. Candan (2018) discovered that among adolescents, “communication skills,” a sub-dimension of social–emotional learning skills, significantly favored female students. However, “problem-solving skills,” “stress coping skills,” and “self-esteem enhancing skills,” other sub-dimensions of social–emotional learning skills, did not significantly differ by gender. Tilki (2021) found a significant gender difference in middle school students’ social–emotional learning skills, with females demonstrating higher skills than males. Similarly, Atın (2022) found that female participants had higher self-awareness and social awareness skills, as well as higher total skill scores, compared to male students, when examining students’ social–emotional learning skills by gender. In contrast, male students exhibited higher communication skills than female students. Studies in the literature consistently indicate that women generally possess higher levels of self-awareness. This finding can be interpreted as women paying more attention to emotional and social processes, thereby enhancing their understanding of internal thoughts (Cross and Madson, 1997; Robins and Trzesniewski, 2005). Conversely, men are often reported to have lower levels of self-awareness and tend to focus on external events (Zhang and Gan, 2015). These gender-based differences provide valuable insights into understanding their implications on individuals’ emotion.

There was no significant difference in social media use between male and female high school students, indicating similar levels of social media use among both genders. This finding is supported by studies in the literature, such as Çakmak (2018), which concluded that there is no significant gender-based difference in students’ levels of social media use. Tutgun Ünal and Deniz (2019) similarly found no significant gender-based difference in students’ levels of social media use. Moreover, multiple studies have indicated that there is no significant difference in levels of social media addiction between genders (Demir Uzun, 2021; Doğrusever, 2021; Yazan, 2021). Many previous studies on social media use and addiction, which included gender-based analyses, have reported differences between male and female participants. According to findings from the study by Güney and Taştepe (2020), girls exhibit higher levels of social media addiction compared to boys. Şişman Eren (2014) found a significant gender difference in the dimension of interpersonal interaction, indicating that boys use social media more for interaction purposes than girls. Atın (2022) examined whether the social media addiction levels of high school students, during the period of extensive social media use in adolescence, varied significantly by gender. The examination concluded that women had significantly higher levels of social media addiction compared to men. In today’s world, where internet usage rates have increased in recent years and gender roles show a flexible structure, it can be said that there is no difference between women and men in the continuous use of social media. Every individual, regardless of gender, is engaged in these channels (Tutgun Ünal and Deniz, 2020).

No significant difference was found in high school students’ social–emotional learning skills across grade levels, except for relationship building skills. A significant difference was found in relationship building skills, favoring 11th-grade students over 10th and 12th-grade students. The 11th-grade students exhibit higher relationship building skills. In general, students across different grade levels show similar social–emotional learning skills. This finding is supported by existing literature. Atın (2022) identified a significant difference between 9th and 10th grade students solely in the self-regulation sub-dimension of social–emotional learning skills. There was no significant difference in grade levels for the other sub-dimensions or the overall scale. Similarly, Kuyulu (2015) found no significant differences in students’ scores on the sub-dimensions of social–emotional learning based on grade level. Accordingly, it was concluded that the grade level variable did not affect the social emotional learning level of the study group. Contrary to our findings, other studies have reported conflicting results. Aksoy (2020) found a statistically significant difference between 12th and 10th graders in the social awareness sub-dimension and the overall scale of the social–emotional learning scale. İşeri (2016) concluded that 9th-grade students have significantly higher levels of social and emotional learning compared to 10th, 11th, and 12th-grade students.

No significant grade-based differences were found in high school students’ social media use levels. Therefore, students from different grades have similar levels of social media use, which is supported by existing literature. Güney and Taştepe (2020) found that social media addiction among adolescents does not significantly vary by grade level. Atın (2022) investigated if the social media addiction levels of high school students vary based on their grade level. After examining the data, it was found that the students’ grade level does not significantly affect their social media addiction levels. Additionally, in many other studies involving high school students, it has been observed that individuals’ levels of social media addiction do not significantly differ based on the grade level they are in (Bilginer, 2020; Deniz and Gürültü, 2018). However, some studies indicate significant differences based on grade levels. For instance, Yazan (2021) identified a notable distinction in social media addiction levels between 9th and 10th grade students, as well as between 9th and 11th grade students. Kalender (2016) found significant differences in social media use habits of high school students depending on grade level. In light of research findings, it can be argued that the reason why adolescents in similar age groups exhibit similar characteristics may be due to the fact that they are in the same developmental period.

## Data Availability

The data analyzed in this study is subject to the following licenses/restrictions: the raw data supporting the conclusions of this article will be made available by the authors, without undue reservation. Requests to access these datasets should be directed to mericeraslan@akdeniz.edu.tr.
